# Psychiatric Diagnosis Revisited: Towards a System of Staging and Profiling Combining Nomothetic and Idiographic Parameters of Momentary Mental States

**DOI:** 10.1371/journal.pone.0059559

**Published:** 2013-03-28

**Authors:** Johanna T. W. Wigman, Jim van Os, Evert Thiery, Catherine Derom, Dina Collip, Nele Jacobs, Marieke Wichers

**Affiliations:** 1 Department of Psychiatry and Psychology, School of Mental Health and Neuroscience, Maastricht University Medical Centre, Maastricht, The Netherlands; 2 Department of Psychiatry, Rob Giel Research Centre, University Medical Centre Groningen, Groningen, The Netherlands; 3 Department of Psychosis Studies, King's College London, Institute of Psychiatry, London, United Kingdom; 4 Department of Neurology, Ghent University Hospital, Ghent, Belgium; 5 Centre of Human Genetics, University Hospital Leuven & Dept of Human Genetics, KU Leuven, Leuven, Belgium; 6 Faculty of Psychology, Open University of the Netherlands, Heerlen, The Netherlands; University of Adelaide, Australia

## Abstract

**Background:**

Mental disorders may be reducible to sets of symptoms, connected through systems of causal relations. A clinical staging model predicts that in earlier stages of illness, symptom expression is both non-specific and diffuse. With illness progression, more specific syndromes emerge. This paper addressed the hypothesis that connection strength and connection variability between mental states differ in the hypothesized direction across different stages of psychopathology.

**Methods:**

In a general population sample of female siblings (mostly twins), the Experience Sampling Method was used to collect repeated measures of three momentary mental states (positive affect, negative affect and paranoia). Staging was operationalized across four levels of increasing severity of psychopathology, based on the total score of the Symptom Check List. Multilevel random regression was used to calculate inter- and intra-mental state connection strength and connection variability over time by modelling each momentary mental state at *t* as a function of the three momentary states at *t*-1, and by examining moderation by SCL-severity.

**Results:**

Mental states impacted dynamically on each other over time, in interaction with SCL-severity groups. Thus, SCL-90 severity groups were characterized by progressively greater inter- and intra-mental state connection strength, and greater inter- and intra-mental state connection variability.

**Conclusion:**

Diagnosis in psychiatry can be described as stages of growing dynamic causal impact of mental states over time. This system achieves a mode of psychiatric diagnosis that combines nomothetic (group-based classification across stages) and idiographic (individual-specific psychopathological profiles) components of psychopathology at the level of momentary mental states impacting on each other over time.

## Introduction

Staging and profiling represent two important aspects of diagnosis in psychiatry, that recently have become more prominent with the work on the newest edition of the widely used classification manual for mental disorders, the Diagnostic and Statistical Manual for Mental disorders (DSM).

### Staging

There is no strong evidence that psychopathology comes as dichotomous "natural types", separated from mental health by ‘zones of rarity’ [1]. Evidence suggests that mental disorders may have dimensional representations, extending from stages of mild behavioural expression of liability in the general population to full-blown clinical psychopathology [1]–[5]. However, the dimensional nature of psychopathology remains to be implemented in psychiatric nosology.

A major question in the construction of the DSM-V was whether a new risk syndrome for psychosis should be added [6], [7]. However, extending only one of the diagnostic categories into its prodromal stage would have resulted in an undesired asymmetry in the diagnostic system, given that all mental disorders have precursor risk states, e.g. depression and bipolar disorder [4], [8]–[11], that furthermore show extensive overlap with each other [12]. As the early, multidimensional expression of psychopathology is non-specific, a more efficient, broad general risk syndrome approach may be required, combining expression of multiple early manifestations of psychopathology rather than creating specific per-disorder risk syndromes [12].

The best example of a model that incorporates the concept of mental illness progressing along stages is the model of clinical staging [13], that aims to ‘move outside the current diagnostic boundaries to include the full spectrum of disorder’ [12] and along dimensional lines links population-level expression of psychopathology to more severe clinical presentations. In the earlier stages of illness, symptom expression is more diffuse, presenting as a ‘general distress’ syndrome; with progression of illness, psychopathological expression becomes more specific and symptoms crystallize more into separate (although overlapping) syndromes [12]. Thus, different stages may be characterized by different mechanisms underlying expression and development of symptoms.

### Profiling

Profiling refers to the tension between the representation of diagnostic categories as homogenous latent classes, used to cluster patients (nomothetic approach), and the widespread observation of heterogeneity between individuals in the same diagnostic class (idiographic approach) that may be important to predict treatment response and outcome. Part of the observed heterogeneity may flow from an incomplete model of psychopathology. For example, it has been proposed that psychopathology may be best conceptualized as a dynamic process of symptoms impacting on each other over time [14]–[16] rather than as latent structures underlying a certain set of clinical symptoms [14]. In this view, mental "disorders" represent sets of symptoms, connected through a system of causal relations. These associations are thought to explain the co-occurrence of different symptoms and are furthermore assumed to span several levels, requiring explanatory pluralism [17]. To examine the notion of symptoms impacting on each other dynamically over time, special methodology collecting frequently repeated measures of mental states underlying symptoms is required, for example the Experience Sampling Method [18].

### Combining Staging and Profiling

**Figure 1:**
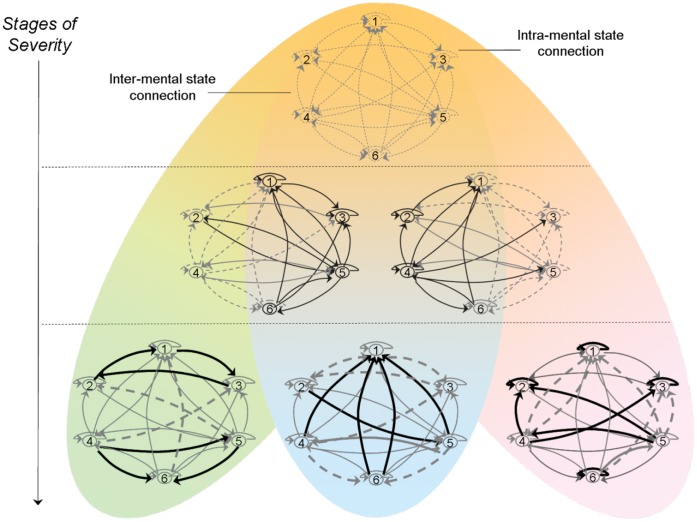
Visual representation of dynamics between mental states with increasing levels of psychopathological severity. Depicted is a hypothesized *circuit* between 6 mental states across different stages of severity. The connections represent the impact of one mental state at time point *t*-1 on another mental state at time-point *t*. The impact of a mental state on itself from *t*-1 to *t* is the intra-mental state connection. In the earliest stage, inter and intra-mental state connections are uniformly weak (top; dotted lines). In the next stage, inter- and intra-mental state connections not only get stronger (middle; solid lines), but there are also more differences between persons with regard to which connections get stronger (person on the left strongest connections on the right side of the circuit, person on the right strongest connections on the left side of the circuit), i.e. there is more profiling. In the most severe stage of psychopathology, mental state connections are growing even stronger (bottom; bold/dotted lines) and more variable across persons. The increased variability in connection strength can be quantified by the random effect from the multilevel random regression model. Thus, staging is represented in this figure by increasing severity of psychopathology, reflected by more powerful connections between mental states impacting on each other, and profiling is represented by the gradual differentiation of syndromes (indicated by different colours) with increasing levels of psychopathological severity. *Based on:* Epskamp, S., Cramer, A. O. J., Waldorp, L. J., Schmittmann, V. D. & Borsboom, D. (2012). Qgraph: Network visualizations of relationships in psychometric data. Journal of Statistical Software, 48, 1–18.

Describing clinical stages may be considered a nomothetic component of diagnosis, whereas the unique symptom profile of patients that move across these classes represents the idiographic part. Since in the earlier stages of illness, symptom expression is more diffuse, presenting as a ‘general distress’ syndrome, whereas with progression of illness, more specific syndromes present themselves [12], it could be hypothesized that the way symptoms impact on each other gradually changes, becoming both stronger and more person-specific with increasing symptom severity. Specifically, it may be hypothesized that in the early stages of illness, associations between symptoms are uniformly weak and do not give rise to high levels of individual-specific profiling in psychopathology. As individuals progress to more severe stages of psychopathology, however, individual-specific associations between symptoms arise, creating a degree of diagnostic divergence (Fig. 1).

In this paper, we examined the hypothesis that the way in which momentary mental states indexing positive affect, negative affect and paranoia impact on each other over time differs across different stages of severity of psychopathology, in that more severe stages of psychopathology are characterized by (i) stronger connections and (ii) more variable connections indicating more individual-specific associations between the mental states over time. The three mental states were chosen as they arguably constitute the core building blocks of an extensive range of symptoms across mental disorders (depression, bipolar disorder, psychosis, anxiety). Psychopathology was assessed and classified across four levels of increasing severity in a general population sample. In order to examine the mutual impact of mental states over time, repeated measures of the three states, collected using the Experience Sampling Method, were used.

We hypothesized that:

- Cross-time associations between mental states indexing positive affect, negative affect and paranoia become stronger across classes of (staged) severity (nomothetic aspect).
- Inter-individual differences in associations between mental states increase with increasing symptom severity, evidenced by an increasing random effect sizes (idiographic aspect).

## Methods

### Sample

This study is part of a larger longitudinal research project investigating the heritability of gene-stress interactions in vulnerability to depression. Data come from 621 general population siblings, sampled from the East Flanders Prospective Twin Study register [19]. The EFPTS is a population-based register, prospectively recording all multiple births in Flanders, Belgium, since 1964. Only women were included in the study, given sex-specific effects in the expression and aetiology of psychopathology. The study was approved by the ethics committee of the Maastricht University Medical Centre and all participants provided written informed consent. The sample was assessed at five time points; the current sample used data from the baseline measurement, when ESM data were collected (see below). Although most subjects were twins, the current study did not require specific twin methodology for analysis.

### Instruments

#### SCL-90

The Symptom Checklist-90-R [20], a reliable and valid screening self-report instrument for a range of symptoms occurring in the past week, was used to index the overall severity of psychopathology with higher scores indicating higher levels of pathology. The SCL-90 consists of nine subscales (Somatization, Obsessive-compulsive, Interpersonal-sensitivity, Depression, Anxiety, Hostility, Phobic anxiety, Paranoid Ideation and Psychoticism), covering the entire range of psychopathology. The total score was divided by its quartiles; the resulting four-level variable (‘*SCL-severity’)* reflected increasing levels of symptom severity.

#### ESM

Data was collected using the Experience Sampling Method, a random time-sampling self-assessment technique that has been shown to be feasible, valid, and reliable [18], [21]. ESM is a structured diary technique that addresses the daily living environment of participants. This method has been described in detail elsewhere [22]. Briefly, participants collected ESM data at 10 random moments on five consecutive days during the day (between 7.30 am and 13.30 pm), providing a maximum of 50 points per person. The semi-random beep-design prevents anticipatory behaviour of participants. Participants were asked to report on thoughts, current context (activity, social context, location), appraisals of the current situation, and affect. Subjects with less than 17 valid reports (out of 50) were excluded.

Three ESM variables reflecting three mental states underlying symptoms across mental disorders [23] were used: negative affect, positive affect and paranoia. Adjectives of mental states were rated by participants on 7-point Likert scales ranging from 1 = ”not at all” to 7 = ”very”. Following earlier work [24], a “negative affect” dimension was constructed based on the mean score of the mood adjectives “insecure, lonely, anxious, guilty and down” (Cronbach’s alpha: 0.76). A “positive affect” dimension was constructed based on the mean score of the mood adjectives “happy”, “enthusiastic”, “energetic” and “satisfied” (Cronbach’s alpha: 0.86). Consistent with earlier work [25], a paranoia score was based on the ESM item “I feel suspicious”, was constructed, also rated on a 7-point Likert scale.

#### SCID

The Structured Clinical Interview for DSM-IV Axis I disorders [26] was administered by trained psychologists to assess levels of clinical symptoms. Subscales of depression, hallucinations and delusions were used for the current study.

#### GAF

A Global Assessment of Functioning score was rated for each participant by an interviewer with a mental health-related profession. The GAF scale has a 100-point range indexing overall mental, social, and occupational functioning of adult individuals. The version with two separate scores was used: a symptom score and a handicap score. Higher scores indicate better functioning.

#### Physical and mental health

Individuals subjectively rated their physical and mental health on a 5-point scale. These variables were recoded into a dichotomous variable, reflecting good (excellent, very good, good) health versus bad (moderate, poor) health.

### Statistical Analyses

All analyses were carried out using STATA 12. The validity of the assumption that the SCL-severity variable represented staged levels of psychopathology and need for care was first investigated. Thus, the four *SCL-severity* groups were compared in relation to the sum score of the depression, hallucination and delusion subscales of the SCID. Similarly, the four *SCL-severity* groups were compared in relation to (i) GAF-symptom and GAF-handicap scores and (ii) subjective ratings of both physical and mental health.

ESM data consist of multiple mental state observations per day, over multiple days, in each person. Analyses were controlled for clustering of data within persons, who, in turn, were clustered within families. As we had no hypothesis regarding genetic effects on transfer of momentary mental states, we did not differentiate between monozygotic and dizygotic twins.

To examine the dynamic impact of the mental states of positive affect, negative affect and paranoia on each other over time, time-lagged variables were used in multilevel random regression models: negative affect at *t* was predicted by (i) negative affect, (ii) positive affect and (iii) paranoia, all at *t*-1. The same was done for positive affect at *t* and paranoia at *t*. Thus, all ESM mental state variables at *t* were predicted by all mental state variables at *t*-1. Each of these analyses was carried out separately in order to enable model convergence.

#### Staging

To examine whether the strength of the associations between mental states over time differed as a function of stages of symptom severity (staging), interactions between mental states (e.g. negative affect at *t*-1) and the four-level SCL-severity variable were fitted in the model predicting the mental state outcome variable (e.g. paranoia at *t*). Using the LINCOM command, regression coefficients (B) were calculated for each SCL-severity group separately. These regression coefficients represent the fixed effects that refer to patterns across groups (i.e. different stages).

#### Profiling

To examine whether more advanced stages of severity of psychopathology were associated with greater level of individual-specific patterns of mental states impacting on each other (profiling), random slopes of mental state predictors at *t*-1 were investigated in the time-lagged analyses described above. These random slopes reflect the assumption that the effect of mental states at *t*-1 predicting mental states at *t* varies randomly across individuals. Random slope effects were estimated for each SCL-severity level to examine the hypothesis of more individual-specific patterns of symptoms impacting on each other with greater levels of psychopathological severity. Additionally, the significance of these differences in random effects was assessed with a simple individual-level linear regression model (SCL-severity group as independent variable and the estimated random slope, predicted with the PREDICT command in STATA, as dependent variable).

## Results

### Sample Characteristics

Of the 621 subjects, 610 participated in the ESM study. Thirty-one participants were excluded because of too little (<30%) valid data points, leaving 579 individuals with data on paranoia and negative affect (268 monozygotic twins, 266 dizygotic twins and 45 non-twin sisters). Data on positive affect was available for 567 individuals. Subjects were aged between 18–61 years (mean age 27.7 (SD 7.9) years). The majority (64%) had a college/university degree, were in a relationship (76%) and employed (62%) or studying (36%) at baseline. Mean level of NA was 1.27 (SD 0.37), of PA 4.43 (SD 0.86), and of momentary paranoia 1.16 (SD 0.33).

### Validation of the SCL-severity Variable

The four *SCL-severity* groups differed in mean levels of delusional ideation (F(3,22672) = 353.3; *p*<0.001), hallucinatory experiences (F(3,22641) = 145.0; *p*<0.001) and depression (F(3,22641) = 1800.0; *p*<0.001). The *SCL-severity* groups similarly differed in GAF symptom score (F(3,22491) = 1157.9; *p*<0.001), GAF handicap score (F(3,22449) = 1185.0; *p*<0.001) and in level of physical (*χ* ^2^(3) = 25000; *p*<0.001) and mental health (*χ* ^2^(3) = 42000; *p*<0.001). Dose-response relations were apparent (Table 1). Thus, the four groups of the *SCL-severity* variable reflect staged levels of symptom severity and impairment.

**Table 1:** Validation of the four quartile groups of the SCL-90 total score as representing increasing levels of symptom severity.

	*SCL-severity* level			
	Level 1	Level 2	Level 3	Level 4
GAF symptom score (mean, SD)	92.4 (5.0)	90.9 (6.0)	88.5 (8.0)	84.3 (11.4)
GAF handicap score (mean, SD)	92.8 (5.0)	91.6 (5.4)	89.9 (6.1)	85.5 (10.3)
Physical health (%)				
Excellent/very good/good	98%	93%	79%	69%
Moderate/poor	2%	7%	21%	31%
Mental health (%)				
Excellent/very good/good	100%	96%	85%	61%
Moderate/poor	0%	4%	15%	39%
SCID delusion score (mean, SD)	0.0 (0.2)	0.1 (0.4)	0.2 (0.5)	0.3 (0.6)
SCID hallucination score (mean, SD)	0 (0)	0.0 (0.1)	0.1 (0.1)	0.1 (0.4)
SCID depression score (mean, SD)	0.6 (0.9)	1.2 (1.2)	1.4 (1.6)	2.7 (2.2)

NB All variables differed significantly across the four levels of SCL-severity (all p<.001).

### Mental States Connection Strength at Different Stages of Symptom Severity

**Figure 2:**
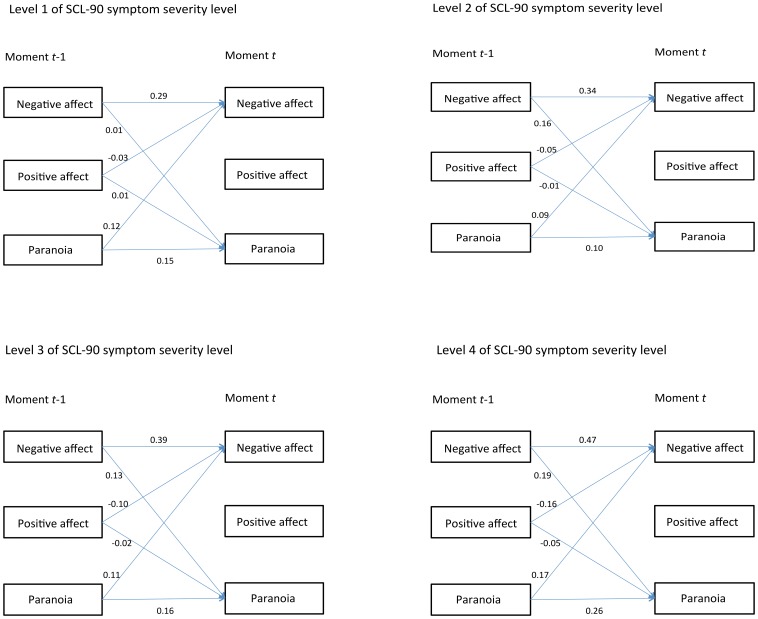
Increasing strength of symptom dynamics with increasing SCL-severity. At the lowest level of SCL-severity (level 1), the regression coefficients of mental states at *t*-1 predicting mental states at *t* are weakest. The strength of these regression coefficients increases in a dose-response fashion with increasing SCL-severity and are strongest at the highest SCL-severity level (level 4).

Significant interactions were found between *SCL-severity* and (i) negative affect at *t*-1 (*Z* = 9.02; *p*<0.001), (ii) positive affect at *t*-1 (*Z* = −12.91; *p*<0.001) and (iii) paranoia at *t*-1 (*Z* = 4.18; *p*<0.001) predicting negative affect at *t* (Table 2). Similarly, significant interactions were found between *SCL-severity* and (i) negative affect at *t*-1 (*Z* = 5.54; *p*<0.001), (ii) positive affect at *t*-1 (*Z* = −5.39; *p*<0.001) and (iii) paranoia at *t*-1 (*Z* = 7.26; *p*<0.001) predicting paranoia at *t*. No significant interactions were found between *SCL-severity* and (i) negative affect at *t*-1 (*Z* = −1.41; *p* = 0.160), (ii) positive affect at *t*-1 (*Z* = −0.64; *p* = 0.522), or (iii) paranoia (*Z* = −1.02; *p* = 0.307) at *t*-1 predicting positive affect at *t*. The significant interactions are displayed in Figure 2, as well as the progressively stronger effect sizes of mental states at *t*-1 impacting on mental states at *t* across the four levels of the *SCL-severity* variable. Thus, mental states at *t*-1 (and in particular negative mental states such as negative affect and paranoia) predict mental states at *t*, suggesting transfer of momentary mental states. This transfer becomes increasingly stronger with increasing symptom severity. This transfer is seen within each mental state (e.g., negative affect at *t*-1 predicts negative affect at *t*) and across mental states (e.g., negative affect at *t*-1 predicts paranoia at *t*).

**Table 2:** Negative affect, positive affect and paranoia at moment *t* predicted by mental states at moment *t*-1, by *SCL-severity.*

Negative affect at moment *t* predicted by mental states at moment *t*-1, by *SCL-severity*			
SCL-Severity	Negative affect at *t*-1	Positive affect at *t*-1	Paranoia at *t-*1
Symptom severity – level 1	0.29 (0.24, 0.33)*	−0.03 (−0.04, 0.01)*	0.12 (0.08, 0.16)*
Symptom severity – level 2	0.34 (0.30, 0.37)*	−0.05 (−0.07, −0.04)*	0.09 (0.06, 0.12)*
Symptom severity – level 3	0.39 (0.36, 0.41)*	−0.10 (−0.12, −0.09)*	0.11 (0.08, 0.14)*
Symptom severity – level 4	0.47 (0.45, 0.49)*	−0.16 (−0.17, −0.14)*	0.17 (0.16, 0.19)*
**Positive affect at moment ** ***t*** ** predicted by mental states at moment ** ***t*** **-1, by ** ***SCL-severity***			
Symptom severity – level 1	−0.26 (−0.35, −0.17)*	0.43 (0.41, 0.46)*	−0.01 (−0.08, 0.06)
Symptom severity – level 2	−0.33 (−0.40, −0.25)*	0.42 (0.39, 0.45)*	−0.10 (−0.17, −0.04)*
Symptom severity – level 3	−0.32 (−0.38, −0.26)*	0.40 (0.37, 0.43)*	−0.10 (−0.15, −0.05)*
Symptom severity – level 4	−0.34 (−0.39, −0.30)*	0.42 (0.40, 0.45)*	−0.08 (−0.12, −0.04)*
**Paranoia at moment ** ***t*** ** predicted by mental states at moment ** ***t*** **-1, by ** ***SCL-severity***			
Symptom severity – level 1	0.01 (−0.05, 0.06)	0.01 (0.00, 0.03)	0.15 (0.11, 0.19)*
Symptom severity – level 2	0.16 (0.12, 0.21)*	−0.01 (−0.03, 0.00)	0.10 (0.06, 0.14)*
Symptom severity – level 3	0.13 (0.10, 0.17)*	−0.02 (−0.03, 0.00)	0.16 (0.13, 0.19)*
Symptom severity – level 4	0.19 (0.17, 0.22)*	−0.05 (−0.07, −0.04)*	0.26 (0.24, 0.28)*

* *p*<0.05.

**Negative affect**: In individuals at the lowest symptom severity level (level 1), negative affect at *t*-1 predicts negative affect at *t.* This association between mental states at two subsequent time points becomes stronger (i.e. there is more transfer) with increasing symptom severity level in a dose-response fashion: this association is strongest at the highest symptom severity level (level 4). Similarly, paranoia affect at *t*-1 predicts negative affect at *t*, and this association also becomes stronger with increasing symptom severity strength, again suggesting more transfer of mental states with increasing symptom severity. Positive affect at *t*-1 predicts negative affect at *t* also with increasing strength, indicating that the lower level of positive affect, the higher the level of negative affect will be, and, given the progressive strength of the association, more transfer of mental states with increasing symptom severity is again suggested.

**Positive affect**: No significant interaction was found for SCL-symptom severity level and mental states at t-1 predicting mental states at t. This is reflected by the fact that there is no clear increase in strength of associations with increasing severity level.

**Paranoia**: In individuals at the lowest symptom severity level (level 1), paranoia at *t*−1 predicts paranoia at *t.* This association between mental states at two subsequent time points becomes stronger (i.e. there is more transfer) with increasing symptom severity level in a dose-response fashion: this association is strongest at the highest symptom severity level (level 4). Similarly, negative affect at *t*−1 predicts paranoia at *t*, and this association also becomes stronger with increasing symptom severity strength, again suggesting more transfer of mental states with increasing symptom severity. Positive affect at *t*−1 predicts paranoia at *t* also with increasing strength, indicating that the lower level of positive affect, the higher the level of paranoia will be, and, given the progressive strength of the association, more transfer of mental states with increasing symptom severity is again suggested.

### Random Effects at Different Stages of Symptom Severity

Random slope effects were examined for the above models with a significant interaction between *SCL-severity* and mental state at *t*-1 (i.e. in the models predicting negative affect and paranoia at *t*). Random slope effects were found to be increasing with increasing levels of *SCL-severity*. These patterns were most clear for negative affect and paranoia (Table 3); random slope effects of positive affect at moment *t*-1 predicting (i) negative affect at moment *t* or (ii) paranoia at moment *t* were very small. Again, a dose-response was apparent, in that random effects were smallest in the lowest *SCL-severity* group of *SCL-severity* and largest in the highest *SCL-severity* group. Thus, larger random effects in the higher *SCL-severity* groups reflect more individual effects, suggesting more profiling with increasing symptom severity.

**Table 3:** Random slope effects in models predicting negative affect and paranoia, by *SCL-severity.*

Random slope effects in models predicting negative affect, by *SCL-severity*			
SCL-severity	Negative affect at *t*-1	Positive affect at *t*-1	Paranoia at *t*-1
Symptom severity – level 1	0.09 (0.08, 0.10)*	0.02 (0.01, 0.03)*	0.10 (0.08, 0.12)*
Symptom severity – level 2	0.10 (0.08, 0.12)*	0.00 (0.00, 0.00)	0.08 (0.06, 0.11)*
Symptom severity – level 3	0.11 (0.09, 0.13)*	0.00 (0.00, 0.00)	0.15 (0.12, 0.19)*
Symptom severity – level 4	0.14 (0.11, 0.17)*	0.05 (0.03, 0.08)*	0.15 (0.11, 0.20)*
**Random slope effects in models predicting paranoia, by ** ***SCL-severity***			
Symptom severity – level 1	0.05 (0.03, 0.10)*	0.04 (0.03, 0.04)*	0.10 (0.08, 0.12)*
Symptom severity – level 2	0.11 (0.09, 0.13)*	0.00 (0.00, 0.00)	0.09 (0.07, 0.12)*
Symptom severity – level 3	0.12 (0.10, 0.15)*	0.01 (0.00, 3.60)	0.14 (0.11, 0.17)*
Symptom severity – level 4	0.20 (0.17, 0.24)*	0.00 (0.00, 0.00)	0.19 (0.15,0.25)*

* *p*<0.05.

The random slope effects reflect variation at the individual level. Thus, the more random effects, the more individual variation is present.

The linear regression analysis predicting the random effects with SCL-severity as independent variable showed that this increase of random effects over the SCL-severity groups (i.e. with increasing symptom severity) was significant for negative affect at *t*-1 (B = 0.06, 95% CI 0.05, 0.07, *p*<0.001) and paranoia at moment *t*-1 (B = 0.02, 95% CI 0.02, 0.03, *p*<0.001) but not for positive affect at moment *t*-1 (B = 0.00, 95% CI 0.00, 0.00, *p*<0.138), predicting negative affect at *t.* Similarly, this increase of random effects over the SCL-severity groups was significant for negative affect at *t*-1 (B = 0.04, 95% CI 0.03, 0.06, *p*<0.001) and paranoia at moment *t*-1 (B = 0.02, 95% CI 0.01, 0.03, *p*<0.001) predicting paranoia at *t*. There was too little variation in the slope of positive affect at *t*-1 predicting paranoia at *t* to predict this based on the *SCL-severity* variable using linear regression.

## Discussion

Mental states indexing momentary positive affect, negative affect and paranoia impacted dynamically on each other over time, and dynamics changed with increasing stages of psychopathological severity in a general population sample of female twins. It was shown that more severe stages of psychopathology are characterized by (i) stronger and (ii) more variable and hence individual-specific inter- and intra-mental state connections over time.

### A Novel Diagnostic System Based on Staging and Profiling?

The results suggest that as individuals move through progressive stages of psychopathological severity as described in the clinical staging model [27], two processes mediate mental health parameters. First, the dynamics between different mental states become increasingly stronger, indicated by the increasing strength of the regression coefficients of mental states at *t*-1 predicting mental states at *t* over the progressive severity stages. Second, the differences between individuals become progressively larger, as indicated by the increasing random slope effects over the progressive severity stages. The first pattern reflects the nomothetic process of staging (progressing through increasingly severe expression of mental ill-health) and the second refers to the idiographic concept of profiling (the increase of inter-individual differences that underlie diagnosable heterogeneity in the expression of psychopathology in clinical populations).

One of the key notions of the clinical staging model is that mental distress and need for care are present long before possible assignment of a clinical diagnosis [12], [27]. As generally recognized, clinical diagnostic classification systems such as the DSM are almost exclusively based on patients whose psychopathology represent the most severe manifestations of mental ill-health [28], [29]. A model of clinical staging acknowledges a need, and a possibility, to intervene before reaching this phase in the course of illness. Therefore, an alternative diagnostic system allowing for flexible operationalization of the nomothetic and idiographic aspects of psychopathology may be productive in this regard. The current study shows that in the context of early diagnosis, nomothetic and idiographic components of psychopathology can be combined, based on the changing dynamics of associations between mental states, in particular negative mental states such as negative affect and paranoia across individuals, and the increasing heterogeneity between individuals, reflected in increasing random (i.e. individual) effects.

### Momentary Mental States, Symptoms and Diagnosis

The symptom measures analysed in the current paper represent mental states indexing momentary positive affect, negative affect and paranoia in daily life. These mental states were measured in the general, mostly non-clinical population, and reflect subclinical, low-level expression of mental ill-health. The dynamics between these mental states may form the building blocks of observable symptoms of mental ill-health and are thus relevant in the context of early diagnosis since it allows for investigation the development of psychopathology arising from its earliest sub-symptomatic expressions.

The rationale explored in this study could be extended to the hypothesis that dynamic patterns can also be observed in actual *symptoms* impacting on each other [14]–[16]. This could be an important and useful addition to the paradigm explored in the current study, complementing the current focus on micro-level momentary mental states with one at the macro-level of symptoms as proposed recently [16]. It is increasingly recognized that symptoms of psychopathology can be described as dynamic networks, or circuits, impacting on each other and crossing diagnostic boundaries [14]–[16]. This hypothesis would be of interest to address in the context of clinical staging, given that staging transgresses diagnostic boundaries [12], and broadly encompasses first expression of mental ill-health in general [27]. The fact that the momentary mental states addressed in the current study have been shown to be relevant in the context of both depression [30] and psychosis [31] supports a spectrum-broad approach: it suggests that emotional dysregulation and psychosis share etiological overlap in their early, non-specific phases of expression and that during the development of more pathological stages, more specific psychopathological profiles arise, based on differentiation of dynamics between mental states [32]–[35].

### Non-linearity Across Stages

When investigating dynamics between mental states (micro-level) or symptoms (macro-level) in the context of development of need for care, the dynamic nature of associations should be taken into account. When moving across the spectrum of psychopathological severity, certain factors of risk or resilience may impact differently at different stages [27], as has recently been shown in the context of psychosis [36]. Also, intermediate phenotypes such as cognitive alterations may show a non-linear pattern of association with expression of psychosis across the full spectrum of the extended psychosis phenotype [37]. Furthermore, non-linear associations also apply to other domains of psychopathology, such as the association between dysregulation of the HPA-axis and depression [38]. This area needs further investigation in future research.

Random effects of positive affect at *t*-1 predicting both negative affect and paranoia at *t*, reflecting individual variation in these associations, were quite small. Thus, effects of positive affect do not differ much between individuals, and these small differences did not change much with increasing symptom severity. Thus, effects of positive affect on negative affect and paranoia are quite similar for all individuals, suggesting interventions that focus on (increasing) positive affect, e.g. mindfulness based interventions [39], that can be implemented relatively easily, may be helpful, independent of diagnosis or symptom severity.

### Strengths and Limitations

An important strength of this study is that it introduces an element of diagnostic novelty, aiming to investigate the development of psychopathology from an entirely new perspective: exploring the dynamics between mental states in individuals with increasing levels of psychopathology. As this is a first, exploratory step in this direction, replication of our findings in other samples is necessary.

It is important to keep in mind that the current sample is a general population sample, in which the expected prevalence of significant psychopathology is around 15–20% [40]. However, mental ill-health is not dichotomous, it is continuously distributed throughout the population [41]–[43], reflecting, at least to a degree, underlying dynamic transitions from one stage to another. The topic of the current paper pertains to longitudinal plasticity of psychopathology, but was examined by analysing a cross-sectional convenience sample. The approach was to analyse between-person differences in severity as a proxy of within-person longitudinal change. The underlying assumptions are that (i) differences in severity of psychopathology in the population reflect, at least to a degree, differences in dynamic transitional trajectories of each participant, rather than differences that are completely stable over the lifetime, and (ii) a random cross-section of the population will contain individuals across the different stages of psychopathology. While this assumption has some face validity, and may be considered suitable for proof-of-principle as demonstrated in the current study, the specific relevance of the present results for the clinical staging model should be considered tentative until replicated in a longitudinal within-person design. Generalization of the current findings should furthermore be conservative, since twins may differ in crucial aspects from the general population. Furthermore, twins were all female, and relatively highly educated.

Future research could extend the line of research explored in the current study with a focus on dynamic relationships between symptoms, comparing dynamics between mental states and/or symptoms across different stages and syndromes to explore and compare possible disorder-specific patterns. Ideally, samples should be used with differential clinical severity patterns, representing multiple clinical stages. Also, studies should ideally include a longitudinal dimension, following individuals over time, to investigate the transition from one stage to another. Furthermore, factors of risk and resilience that may impact on mental state and symptom dynamics, and on progression through successive clinical stages, plus their susceptibility to early interventions [44], should be studied in the future.

## References

[1] Kendell R, Jablensky A (2003) Distinguishing between the validity and utility of psychiatric diagnoses. Am J Psychiatry 160(1): 4–12. 12505793 10.1176/appi.ajp.160.1.4

[2] Haslam N, Holland E, Kuppens P (2011) Categories versus dimensions in personality and psychopathology: A quantitative review of taxometric research. Psychol Med1(1): 1–18. 10.1017/S0033291711001966 21939592

[3] Kendler KS, Gardner CO (1998) Boundaries of major depression: An evaluation of DSM-IV criteria. Am J Psychiatry 155: 172–7. 9464194 10.1176/ajp.155.2.172

[4] Angst J, Gamma A, Benazzi F, Ajdacic V, Eich D, et al (2003) Toward a re-definition of subthreshold bipolarity: epidemiology and proposed criteria for bipolar-II, minor bipolar disorders and hypomania. J Affect Disord 73(1–2): 133–46. 12507746 10.1016/s0165-0327(02)00322-1

[5] Krueger RF, Piasecki TM (2002) Toward a dimensional and psychometrically-informed approach to conceptualizing psychopathology. Behav Res Ther 40(5): 485–99. 12038642 10.1016/s0005-7967(02)00016-5

[6] Carpenter WT, van Os J (2011) Should attenuated psychosis syndrome be a DSM-5 diagnosis? Am J Psychiatry 168(5): 460–3. 21536700 10.1176/appi.ajp.2011.10121816

[7] Fusar-Poli P, Yung AR (2012) Should attenuated psychosis syndrome be included in DSM-5? Lancet 379(9816): 591–2. 22340290 10.1016/S0140-6736(11)61507-9

[8] Fava GA, Grandi S, Canestrari R, Molnar G (1990) Prodromal symptoms in primary major depressive disorder. J Affect Disord 19(2): 149–52. 2142701 10.1016/0165-0327(90)90020-9

[9] Jackson A, Cavanagh J, Scott J (2003) A systematic review of manic and depressive prodromes. J Affect Disord 74(3): 209–17. 12738039 10.1016/s0165-0327(02)00266-5

[10] Regeer EJ, Krabbendam L, de Graaf R, ten Have M, Nolen WA, et al (2006) A prospective study of the transition rates of subthreshold (hypo)mania and depression in the general population. Psychol Med 36(5): 619–27. 16438739 10.1017/S0033291705006823

[11] Tijssen MJ, van Os J, Wittchen HU, Lieb R, Beesdo K, et al (2010) Evidence that bipolar disorder is the poor outcome fraction of a common developmental phenotype: an 8-year cohort study in young people. Psychol Med 40(2): 289–99. 19515266 10.1017/S0033291709006138

[12] McGorry P, Van Os J (2013) Redeeming Diagnosis in Psychiatry: Timing versus Specificity. Lancet 381(9863): 343–345. 23351805 10.1016/S0140-6736(12)61268-9

[13] McGorry PD, Hickie IB, Yung AR, Pantelis C, Jackson HJ (2006) Clinical staging of psychiatric disorders: a heuristic framework for choosing earlier, safer and more effective interventions. Aust NZ J Psychiat 40(8): 616–22. 10.1080/j.1440-1614.2006.01860.x 16866756

[14] Kendler K, Zachar P, Craver C (2011) What kinds of things are psychiatric disorders? Psychol Med 41(6): 1143. 20860872 10.1017/S0033291710001844

[15] Borsboom D, Cramer AOJ, Schmittmann VD, Epskamp S, Waldorp LJ (2011) The small world of psychopathology. PloS one 6(11): e27407. 22114671 10.1371/journal.pone.0027407 PMC3219664

[16] van Os J, Linscott RJ (2012) Introduction: The extended psychosis phenotype–relationship with schizophrenia and with ultrahigh risk status for psychosis. Schizophr Bull 38(2): 227–30. 22355185 10.1093/schbul/sbr188 PMC3283160

[17] Kendler KS (2005) Toward a philosophical structure for psychiatry. Am J Psychiatry 162(3): 433–40. 15741457 10.1176/appi.ajp.162.3.433

[18] Myin-Germeys I, Oorschot M, Collip D, Lataster J, Delespaul P, et al (2009) Experience sampling research in psychopathology: opening the black box of daily life. Psychol Med 39(09): 1533–47. 19215626 10.1017/S0033291708004947

[19] Derom CA, Vlietinck RF, Thiery EW, Leroy FO, Fryns JP, et al (2006) The East Flanders Prospective Twin Survey (EFPTS). Twin Res Hum Genet 9(6): 733–8. 17254399 10.1375/183242706779462723

[20] Derogatis LR (1977) SCL-90. Administration, scoring & procedures manual-I for the (revised) version and other instruments of the psychopathology rating scale series. Baltimore, MD: Clinical Psychometrics Research Unit, Johns Hopkins University School of Medicine.

[21] Csikszentmihalyi M, Larson R (1987) Validity and reliability of the Experience-Sampling Method. J Nerv Ment Dis 175(9): 526–36. 3655778 10.1097/00005053-198709000-00004

[22] Wichers M, Myin-Germeys I, Jacobs N, Peeters F, Kenis G, et al (2007) Genetic risk of depression and stress-induced negative affect in daily life. Br J Psychiatry 191: 218–23. 17766761 10.1192/bjp.bp.106.032201

[23] Myin-Germeys I, Peeters F, Havermans R, Nicolson NA, DeVries MW, et al (2003) Emotional reactivity to daily life stress in psychosis and affective disorder: an experience sampling study. Acta Psychiatr Scand 107(2): 124–31. 12534438 10.1034/j.1600-0447.2003.02025.x

[24] Myin-Germeys I, Van Os J, Schwartz JE, Stone AA, Delespaul PA (2001) Emotional reactivity to daily life stress in psychosis. Arch Gen Psychiatry 58(12): 1137–44. 11735842 10.1001/archpsyc.58.12.1137

[25] Simons CJ, Wichers M, Derom C, Thiery E, Myin-Germeys I, et al (2009) Subtle gene-environment interactions driving paranoia in daily life. Genes Brain Behav 8(1): 5–12. 18721261 10.1111/j.1601-183X.2008.00434.x

[26] First MB, Gibbon M (1997) User's guide for the Structured clinical interview for DSM-IV axis I disorders SCID-I: clinician version. American Psychiatric Pub Incorporated.

[27] McGorry PD, Killackey E, Yung AR (2007) Early intervention in psychotic disorders: detection and treatment of the first episode and the critical early stages. Med J Aust 187(7 Suppl): S8–10. 10.5694/j.1326-5377.2007.tb01327.x 17908033

[28] Maric N, Myin-Germeys I, Delespaul P, De Graaf R, Vollebergh W, et al (2004) Is our concept of schizophrenia influenced by Berkson's bias? Soc Psychiatry Psychiatr Epidemiol 39(8): 600–5. 15300369 10.1007/s00127-004-0803-z

[29] Regeer EJ, Krabbendam L, De Graaf R, Have MT, Nolen WA, et al (2009) Berkson's bias and the mood dimensions of bipolar disorder. Int J Methods Psychiatr Res 18(4): 279–86. 19708034 10.1002/mpr.290 PMC6878283

[30] Wichers M, Lothmann C, Simons CJP, Nicolson NA, Peeters F (2011) The dynamic interplay between negative and positive emotions in daily life predicts response to treatment in depression: A momentary assessment study. Brit J Clin Psychol 51(2): 206–22. 22574805 10.1111/j.2044-8260.2011.02021.x

[31] Kramer IM, Simons CJ, Myin-Germeys I, Jacobs N, Derom C, et al (2011) Evidence that genes for depression impact on the pathway from trauma to psychotic-like symptoms by occasioning emotional dysregulation. Psychol Med 42(2): 283–294. 21835094 10.1017/S0033291711001474

[32] van Rossum I, Dominguez MD, Lieb R, Wittchen HU, van Os J (2011) Affective dysregulation and reality distortion: a 10-year prospective study of their association and clinical relevance. Schizophr Bull 37(3): 561–71. 19793794 10.1093/schbul/sbp101 PMC3080695

[33] Wigman JT, van Nierop M, Vollebergh WA, Lieb R, Beesdo-Baum K, et al.. (2012) Evidence That Psychotic Symptoms Are Prevalent in Disorders of Anxiety and Depression, Impacting on Illness Onset, Risk, and Severity–Implications for Diagnosis and Ultra-High Risk Research. Schizophr Bull DOI sbr196 (pii)10.1093/schbul/sbr196. 10.1093/schbul/sbr196 PMC3283146 22258882

[34] Green C, Garety PA, Freeman D, Fowler D, Bebbington P, et al (2006) Content and affect in persecutory delusions. Br J Clin Psychol 45(Pt 4): 561–77. 10.1348/014466506X98768 17076964

[35] Freeman D (2007) Suspicious minds: the psychology of persecutory delusions. Clin Psychol Rev 27(4): 425–57. 17258852 10.1016/j.cpr.2006.10.004

[36] Binbay T, Drukker M, Elbi H, Aksu Tanık F, Özkınay F, et al (2012) Testing the Psychosis Continuum: Differential Impact of Genetic and Nongenetic Risk Factors and Comorbid Psychopathology Across the Entire Spectrum of Psychosis. Schizophr Bull 38(5): 992–1002. 21525167 10.1093/schbul/sbr003 PMC3446240

[37] Kravariti E, Russo M, Vassos E, Morgan K, Fearon P, et al (2012) Linear and non-linear associations of symptom dimensions and cognitive function in first-onset psychosis. Schizophr Res 140(1–3): 221–231. 22766128 10.1016/j.schres.2012.06.008

[38] Wardenaar KJ, Vreeburg SA, van Veen T, Giltay EJ, Veen G, et al (2011) Dimensions of depression and anxiety and the hypothalamo-pituitary-adrenal axis. Biol Psychiat 69(4): 366–73. 21030010 10.1016/j.biopsych.2010.09.005

[39] Chiesa A, Serretti A (2011) Mindfulness based cognitive therapy for psychiatric disorders: a systematic review and meta-analysis. Psychiat Res 187(3): 441–53. 10.1016/j.psychres.2010.08.011 20846726

[40] Wittchen H, Jacobi F, Rehm J, Gustavsson A, Svensson M, et al (2011) The size and burden of mental disorders and other disorders of the brain in Europe 2010. Eur Neuropsychopharm 21(9): 655–79. 10.1016/j.euroneuro.2011.07.018 21896369

[41] Anderson J, Huppert F, Rose G (1993) Normality, deviance and minor psychiatric morbidity in the community. Psychol Med 23(02): 475–85. 8332661 10.1017/s0033291700028567

[42] Goldberg D (2000) Plato versus Aristotle: categorical and dimensional models for common mental disorders. Compr Psychiat 41(2): 8–13. 10746898 10.1016/s0010-440x(00)80002-4

[43] Whittington JE, Huppert FA (1996) Changes in the prevalence of psychiatric disorder in a community are related to changes in the mean level of psychiatric symptoms. Psychol Med 26(6): 1253–60. 8931171 10.1017/s0033291700035972

[44] McGorry P (2007) Issues for DSM-V: clinical staging: a heuristic pathway to valid nosology and safer, more effective treatment in psychiatry. Am J Psychiatry 164(6): 859–60. 17541042 10.1176/ajp.2007.164.6.859

